# The preference choices of *Conopomorpha sinensis* Bradley (Lepidoptera: Gracilariidae) for litchi based on its host surface characteristics and volatiles

**DOI:** 10.1038/s41598-018-20383-4

**Published:** 2018-01-31

**Authors:** Xiang Meng, Junjie Hu, Yanhua Li, Jianqing Dai, Mingfang Guo, Gecheng Ouyang

**Affiliations:** 1 Guangdong Key Laboratory of Animal Conservation and Resource Utilization, Guangdong Public Laboratory of Wild Animal Conservation and Utilization, Guangdong Institute of Applied Biological Resources, Guangzhou, 510260 China; 2 College of Life Science, Guangzhou University, Guangzhou, 510006 China

## Abstract

*Conopomorpha sinensis* Bradley is a host-specific pest of *Litchi chinensis* and *Euphoria longan*. Here, we demonstrated that *C. sinensis* has evolved special physical and chemical mechanisms for host plant location that enable it to survive and reproduce. Females favored laying their eggs on the convex surface of litchi fruit that had particular volatile characteristics. Experiments using a H-type olfactometer showed that female *C. sinensis* were attracted to litchi flowers, tender shoots, immature fruits, and mature fruits, with the highest attraction rate to mature fruits (74.67 ± 2.31%). There were no significant differences in the attraction of male *C. sinensis* to different litchi tissues. Further oviposition preference tests using the pericarp, pulp, and seeds of mature litchi fruits revealed that female *C. sinensis* prefer to lay their eggs on the pericarp. Litchi volatiles were found to be important in attracting *C. sinensis* to fruits for oviposition. Analysis of volatiles from different litchi tissues by HS-SPME-GC-MS revealed 31 similar volatiles, some of which may be important in the oviposition preference choices of *C. sinensis* on litchi fruit.

## Introduction

*Conopomorpha sinensis* Bradley (Lepidoptera: Gracilariidae) is an important pest of *Litchi chinensis* and *Euphoria longan*^1^. Its larvae can bore into litchi fruit, damaging flowers, tender shoots, and leaves. After hatching, the larvae immediately penetrate the fruits, feeding on the seed neck, and can transfer a variety of microorganisms that infect the fruits and eventually destroy the crop^2,3^. This has caused severe economic loss to the Chinese litchi industry, with disastrous consequences for the export of Litchi and longan fruit^4^. Due to its cryptic feeding behavior and overlapping of generations, *C. sinensis* is difficult to control. Difficulties experienced with chemical control have led researchers to explore the use of biological and molecular approaches to controlling this pest^5–7^.

Although chemicals and physical irradiation can control *C. sinensis*^8–10^, biological control, which is one of the most widely used environmentally benign approaches to controlling pests^11^, has focused on the natural enemies of *C. sinensis*. These include many species of spider, green lacewings, predatory mirids, ladybugs, ants, and parasitic wasps^6,12^. Plant protectants and biopesticides can also control *C. sinensis*^13,14^. Techniques based on chemical ecology have received less attention, except for primary screens of plant volatiles to repel or lure *C. sinensis*^15–18^. Whether the attraction of *C. sinensis* to litchi is mediated via behavior and chemoreception is unknown.

The selection and adaptation of insects to their hosts are the result of co-evolution^19–22^. Both the highly sensitive olfactory system of the insect and the odor of the host plant play vital roles in insect survival and reproduction^23^. Studying the mechanisms underlying chemical communication between insects and plants can provide a target for the development of highly efficient and specific regulators of insect behavior^24^. The aims of the present study were to identify the factors responsible for the selection of litchi as a suitable host by *C. sinensis*, and to screen for host volatiles capable of luring *C. sinensis*. This study will be useful in the development of insect attractants for field biological control and forecasting insect outbreaks. A better understanding of the chemical ecology of litchi and *C. sinensis* will help to provide a green biocontrol technology with the characteristics of non-toxicity, non-pollution, and sustainability.

## Results

### The choice behavior of *C. sinensis* depends on the surface characteristics and volatiles of host plants and non-host plants

**Figure 1:**
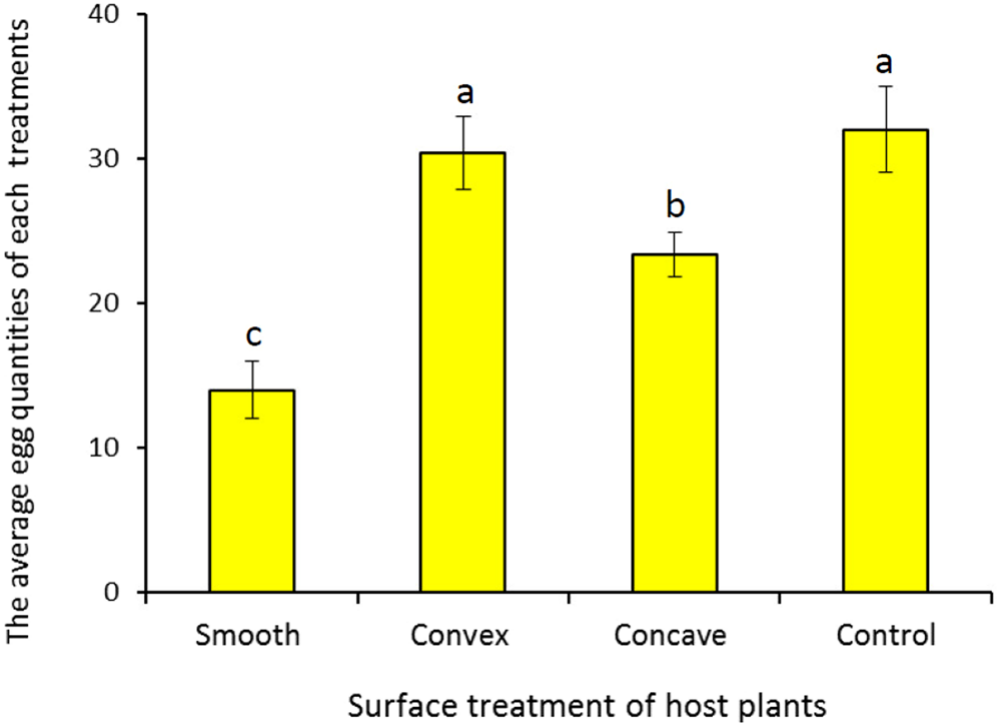
The choice behavior of *C. sinensis* to the surface characteristics of host plants. Different letters above the columns indicate significant differences in behavior of *C. sinensis* to different treatments (*P* < 0.05).

**Figure 2:**
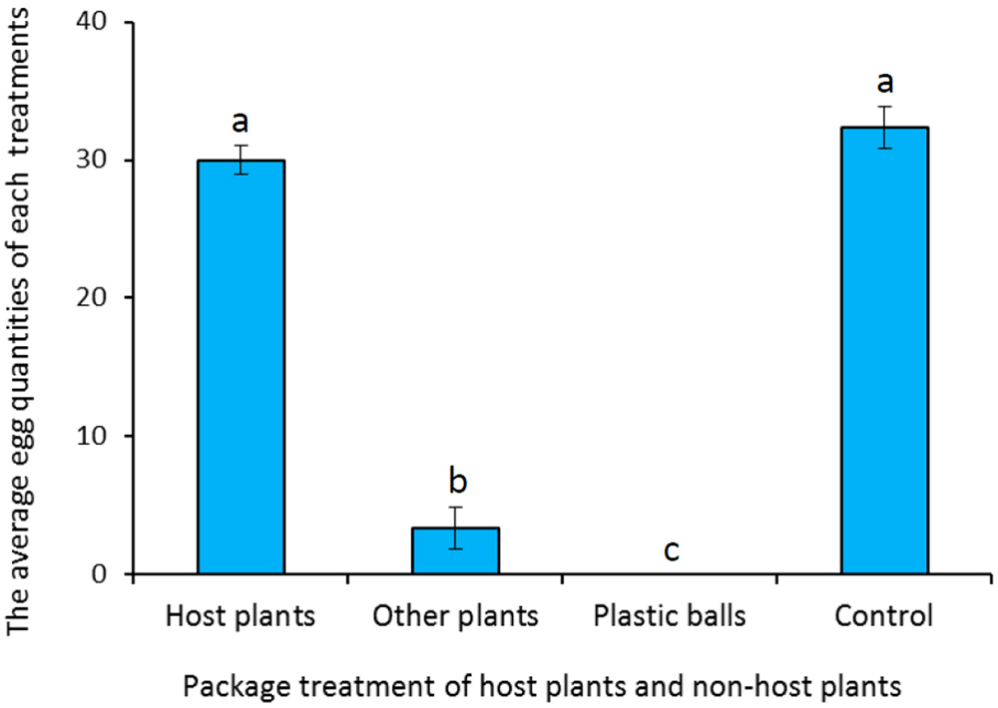
The choice behavior of *C. sinensis* to host plants and non-host plants. Different letters above the columns indicate significant differences in behavior of *C. sinensis* to different treatments (*P* < 0.05).

The taxis behavior response of *C. sinensis* to the surface of host plants and differences in their responses to host plants and non-hosts were observed. There were significant differences in oviposition choice by female *C. sinensis* on different surfaces of host plants, which preferred to oviposit on the convex surface of host plants (*F* = 37.14, *df* = 3, 12, *P* < 0.05). The average number of eggs deposited on the convex surface of host plants was 30.33 ± 2.52, which was similar to that of the litchi fruit control (32.00 ± 2.00) (Fig. 1). The second favorite oviposition site was the concave surface of host plants. Female *C. sinensis* preferred host plants to non-host plants (*F* = 20.14, *df* = 3, 12, *P* < 0.05), which was similar to that of the control group (Fig. 2). Otherwise, there were fewer eggs, and even no difference in oviposition between non-host plants and non-host plastic balls. This demonstrates that the surface physical characteristics and volatiles of litchi had effects on the oviposition of *C. sinensis*.

### Taxis reactions of *C. sinensis* to different tissues of litchi

In this study, the taxis behavior of adult *C. sinensis* to different litchi tissues was studied using a H-type olfactometer^13,25^. The results showed differences in the taxis behavior of female and male adults of *C. sinensis* to different tissues of the litchi host (Table 1), with female adults preferring the host more than male adults. The attraction rates of mature fruits to female adult *C. sinensis* was 74.67 ± 2.31%, which had a significant attraction than other tissues of host plants (*F* = 44.98, *df* = 3, 12, *P* < 0.05). The attraction rate of male adult *C. sinensis* to different litchi tissues was less than 27%, although this was not statistically significant (*F* = 4.44, *df* = 3, 12, *P* > 0.05).

**Table 1:** Taxis reaction analysis of *C. sinensis* to different tissues of litchi.

Adult *C. sinensis*	Litchi tissue	Number of insects in sample	Average number showing taxis reaction		Attraction rate
			Treatment	CK	
female	flower	50	32.00	18.00	28.00 ± 4.00% D
	tender shoots	50	38.33	11.67	53.33 ± 8.33% B
	immature fruit	50	35.00	15.00	40.00 ± 4.00% C
	mature fruit	50	43.67	6.33	74.67 ± 2.31% A
male	flower	50	27.33	22.67	9.33 ± 2.31% ab
	tender shoots	50	29.67	20.33	18.67 ± 4.62% a
	immature fruit	50	26.33	23.67	5.33 ± 6.11% b
	mature fruit	50	28.67	18.00	26.67 ± 6.43% a

Capital letters above the columns indicate significant differences in the taxis reaction of female *C. sinensis* to different tissues of litchi (p < 0.05). Small letters above the columns indicate significant differences in the taxis reaction of female *C. sinensis* to different tissues of litchi (p < 0.05).

### Oviposition preference of female *C. sinensis* to different tissues of litchi fruit

**Figure 3:**
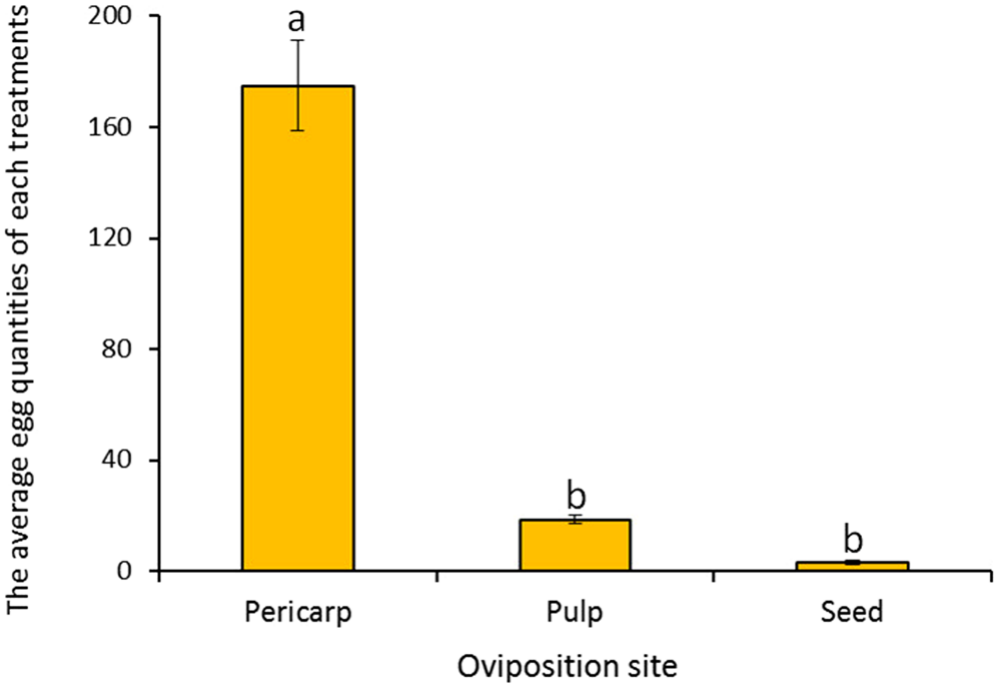
Oviposition preference analysis of female *C. sinensis* to different tissues of litchi fruit. Different letters above the columns indicate significant differences in oviposition preference of female *C. sinensis* to different tissues of litchi fruit (*P* < 0.05).

The oviposition preference test showed that the number and proportion of laid eggs on pericarp tissues were the largest among all the treatments (Fig. 3). Female adults laid 175 ± 28 eggs on average and 88.83% in total on the pericarp, which is significantly different to the values obtained with pulps and seeds of litchi fruit (*F* = 10.93, *df* = 3, 9, *P* < 0.05).

### Isolation and identification of volatiles from different litchi tissues

**Figure 4:**
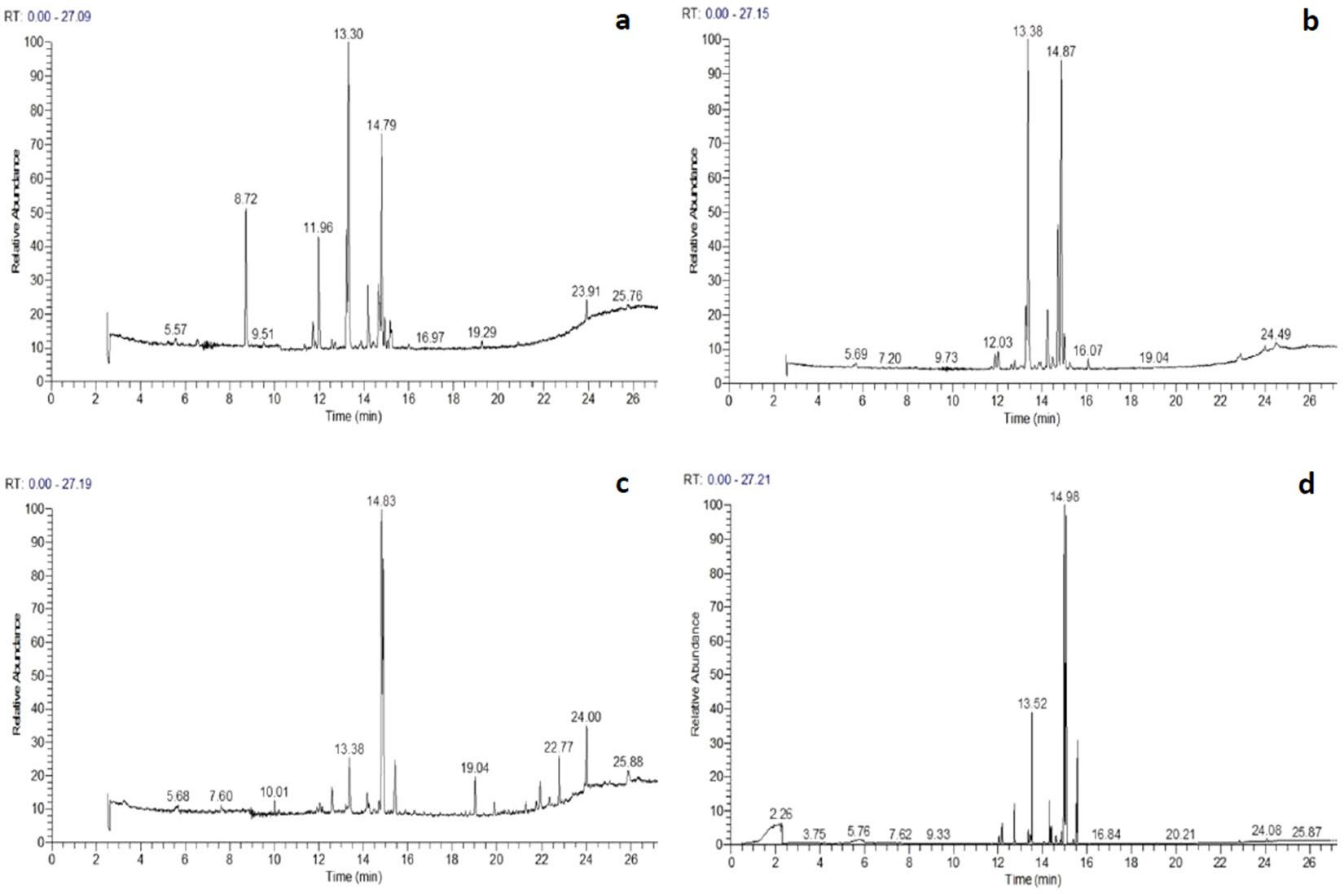
HS-SPME-GC-MS chromatogram of volatiles from different litchi tissues. (**a**) flower (**b**) tender shoots (**c**) immature fruit (**d**) mature fruit.

Results of HS-SPME-GC-MS analysis indicated that the volatiles of different litchi tissues were diverse. Figure 4 shows representative chromatograms of litchi: (a) flowers, (b) tender shoots, (c) immature fruits, and (d) mature fruits. The numbers of chromatographic peaks for each tissue type were 17, 15, 12, and 28, respectively, and 42 volatile compounds were identified. A database comparison of mass spectra revealed 31 similar volatiles (Similarity, SI >800; Table 2). The individual chemical compounds and their percentage in the volatile emissions of different litchi tissues are shown in Table 2. The analysis showed that the major compounds of the volatiles from different litchi tissues were terpenes (21%), alcohols (3%), and esters (2%). There were also large differences among the volatiles: litchi flowers contained caryophyllene (25.11%), (+)-aromadendrene (18.70%), α-copaene (10.76%); volatiles from tender shoots contained ß-elemene (38.7%), (+)-aromadendrene (29.18%), (+)-epi-bicyclosesquiphellandrene (13.48%); immature fruit contained mainly 1,3-cyclohexadiene, 5-(1,5-dimethyl-4-hexenyl)−2-methyl-, [S-(R*,S*)]- (39.01%), l-ß-bisabolene (27.06%), benzene, 1-(1,5-dimethyl-4-hexenyl)−4-methyl- (9.02%); and mature fruit contained mainly 1,3-cyclohexadiene, 5-(1,5-dimethyl-4-hexenyl)−2-methyl-, [S-(R*,S*)]- (35.21%), l-ß-bisabolene (27.75%), and caryophyllene (9.71%). The volatile common to the different litchi tissues was (+)-aromadendrene, which was found in the following proportions: tender shoots (29.18%)> flowers (18.70%)> immature fruits (8.41%)> mature fruits (0.59%).

**Table 2:** Relative contents of volatile compounds detected from different litchi tissues.

No.	CAS	Compounds	Molecular formula	Relative content			
				F	TS	IF	MF
1	1066-40-6	Silanol, trimethyl-	C_3_H_10_OSi	ND	ND	ND	4.24
2	3856-25-5	α-Copaene	C_15_H_24_	10.76	1.69	ND	3.03
3	489-39-4	(+)-Aromadendrene	C_15_H_24_	18.70	29.18	8.41	0.59
4	88-84-6	ß-Guaiene	C_15_H_24_	ND	ND	ND	0.05
5	11094-59-0	Docosahexaenoic acid, 1,2,3-propanetriyl ester	C_69_H_101_O_6_	ND	0.77	3.00	ND
6	5937-11-1	T-Cadinol	C_15_H_26_O	ND	3.01	ND	ND
7	22393-88-0	Oleic acid, eicosyl ester	C_38_H_74_O_2_	ND	ND	3.01	ND
8	13744-15-5	1H-Cyclopenta[1,3]cyclopropa[1,2]benzene, octahydro-7-methyl-3-methylene-4-(1-methylethyl)-, [3aS-(3aà,3bá,4á,7à,7aS*)]-	C_15_H_24_	6.26	ND	ND	ND
9	10219-75-7	Naphthalene, 1,2,3,5,6,7,8,8a-octahydro-1,8a-dimethyl-7-(1-methylethenyl)-, [1S-(1à,7à,8aà)]-	C_15_H_24_	7.64	ND	ND	ND
10	PubChem CID: 5368784	1,4,7,-Cycloundecatriene, 1,5,9,9-tetramethyl-, Z,Z,Z-	C_15_H_24_	5.03	ND	ND	ND
11	1253-84-5	Cholestane-3,5,6-triol,(3b,5a,6b)-	C_27_H_48_O_3_	1.02	ND	ND	ND
12	22469-52-9	(+)-Cyclosativene	C_15_H_24_	ND	ND	ND	0.77
13	11028-42-5	Cedrene	C_15_H_24_	ND	1.50	ND	1.69
14	73744-93-1	(+)-Epi-bicyclosesquiphellandrene	C_15_H_24_	ND	13.48	ND	2.58
15	19891-74-8	Lycoxanthin	C_40_H_56_O	ND	ND	3.09	ND
16	17699-14-8	α-Cubebene	C_15_H_24_	8.53	ND	ND	0.07
17	13474-59-4	α-trans-Bergamotene	C_15_H_24_	ND	ND	ND	0.88
18	150320-52-8	2-Isopropyl-5-methyl-9-methylenebicyclo[4.4.0]dec-1-ene	C_15_H_24_	ND	ND	ND	0.92
19	514-51-2	ß-Patchoulene	C_15_H_24_	ND	0.73	ND	0.07
20	52617-34-2	Seychellene	C_15_H_24_	2.40	1.49	ND	ND
21	6753-98-6	Isocaryophillene	C_15_H_24_	ND	ND	ND	3.31
22	18794-84-8	ß-Farnesene	C_15_H_24_	10.04	ND	ND	1.84
23	87-44-5	Caryophyllene	C_15_H_24_	25.11	6.51	ND	9.71
24	110-83-8	Cyclohexene	C_6_H_10_	ND	ND	ND	0.18
25	158848-19-2	7-Epizingiberene	C_15_H_24_	ND	ND	39.01	35.21
26	495-61-4	l-ß-Bisabolene	C_15_H_24_	ND	ND	27.06	27.75
27	19870-75-8	Cedrane, 8-propoxy-	C_18_H_32_O	ND	ND	4.52	ND
28	33880-83-0	ß-Elemene	C_15_H_24_	ND	38.7	ND	0.10
29	2306-78-7	Nerolidyl acetate	C_17_H_28_O_2_	ND	ND	ND	0.08
30	644-30-4	Benzene, 1-(1,5-dimethyl-4-hexenyl)−4-methyl-	C_15_H_22_	ND	ND	9.02	6.30
31	483-76-1	d-Cadinene	C_15_H_24_	2.74	ND	ND	0.35

ND: not detected; F: flower; TS: tender shoots; IF: immature fruit; MF: mature fruit. No. 10: no CAS number.

## Discussion

The relationship between insects and plants is mainly a reflection of insect feeding, oviposition, and choice of habitat^26^. The host range of *C. sinensis* is narrow, and there is high host specificity. To date, *C. sinensis* has only been reported in *Litchi chinensis* and *Euphoria longan* in South China and Southeast Asia^6,27,28^. Previous studies show that the cultivar of *Litchi chinensis* most seriously affected by *C. sinensis* is Feizixiao^15^. In the present study, physical and chemical choice behavior tests confirmed that *C. sinensis* exhibited a preference for different tissues of the litchi cultivar Feizixiao. The host choice behavior of *C. sinensis* showed that female *C. sinensis* preferred to oviposit on a convex surface that resembled that of the surface of litchi fruit. Some researchers link this oviposition behavior to predator avoidance^29–31^. In addition, differences in the selection of host plants and non-hosts by *C. sinensis* confirmed that host volatiles attract *C. sinensis* to the oviposit. For many herbivorous insects, host plant selection is very important for progeny survival and fitness^32^.

The relationship between herbivorous insects and host plant compounds plays a vital role in the preference choices of insects to their host plants^33–35^. Different tissues and volatile cues from host plants are crucial for insect attraction, affecting choice of feeding sites^36^, oviposition behavior^37^, helping offspring to locate and recognize a suitable host plant for larval growth and movement^38,39^, and even finding a mating partner^40^. Behavioral assays in a H-type olfactometer showed that adult female *C. sinensis* preferred mature fruits to the other tissues of host plants. By contrast, there were no significant differences in the taxis reactions of male adult *C. sinensis* among the different litchi tissues. This suggests that host plant compounds and their nutritional quality might be key factors in offspring survival^30,41–43^. Tests of the oviposition preference of female *C. sinensis* to different tissues of mature litchi fruits indicated that the greatest number and proportion of eggs were laid on the pericarp. This is consistent with the results of Xian *et al*.^13^, who reported that *C. sinensis* preferred the pericarp of host plant fruits after mating.

Plants can produce complicated odor blends^44^, with large numbers of volatiles comprising several compounds in particular ratios. These volatiles strongly influence the ecological interactions of plants with insects^45^. In the present study, 42 compounds, many of which were terpenes, were identified in the volatile blend emitted from different litchi tissues (Table 2). These findings provide a theoretical basis for studying chemical communication between *C. sinensis* and litchi. Previous studies revealed that zingiberene attracts *C. sinensis* to lay eggs on its host^18^. (E)-ß-farnesene and (Z)-ß-farnesene were two unique compounds from mature fruit pericarp of the litchi cultivar Feizixiao, and it was speculated that specific ratios of these compounds and six other volatiles that were mainly found in representative susceptible cultivars of litchi may contribute to the attraction of *C. sinensis*^46^. Our results suggest that the chemical composition of mature litchi fruit is most conducive to successful attraction of adult female *C. sinensis*. We found that silanol, trimethyl-, 2-isopropyl-5-methyl-9-methylenebicyclo[4.4.0]dec-1-ene, isocaryophillene, α-trans-bergamotene, (+)-cyclosativene, and cyclohexene were unique compounds of the volatiles of mature Feizixiao fruit. In addition, 7-epizingiberene, l-ß-bisabolene, and caryophyllene were detected as the main compounds of the volatiles emitted from immature and mature fruit, while (+)-aromadendrene is the common compound of the volatiles from different litchi tissues. Volatile blends comprising these constituents in specific proportions may be key signals for host identification in *C. sinensis*. How the mature fruit processes such volatile mixtures is unclear and requires further research.

**Figure 5:**
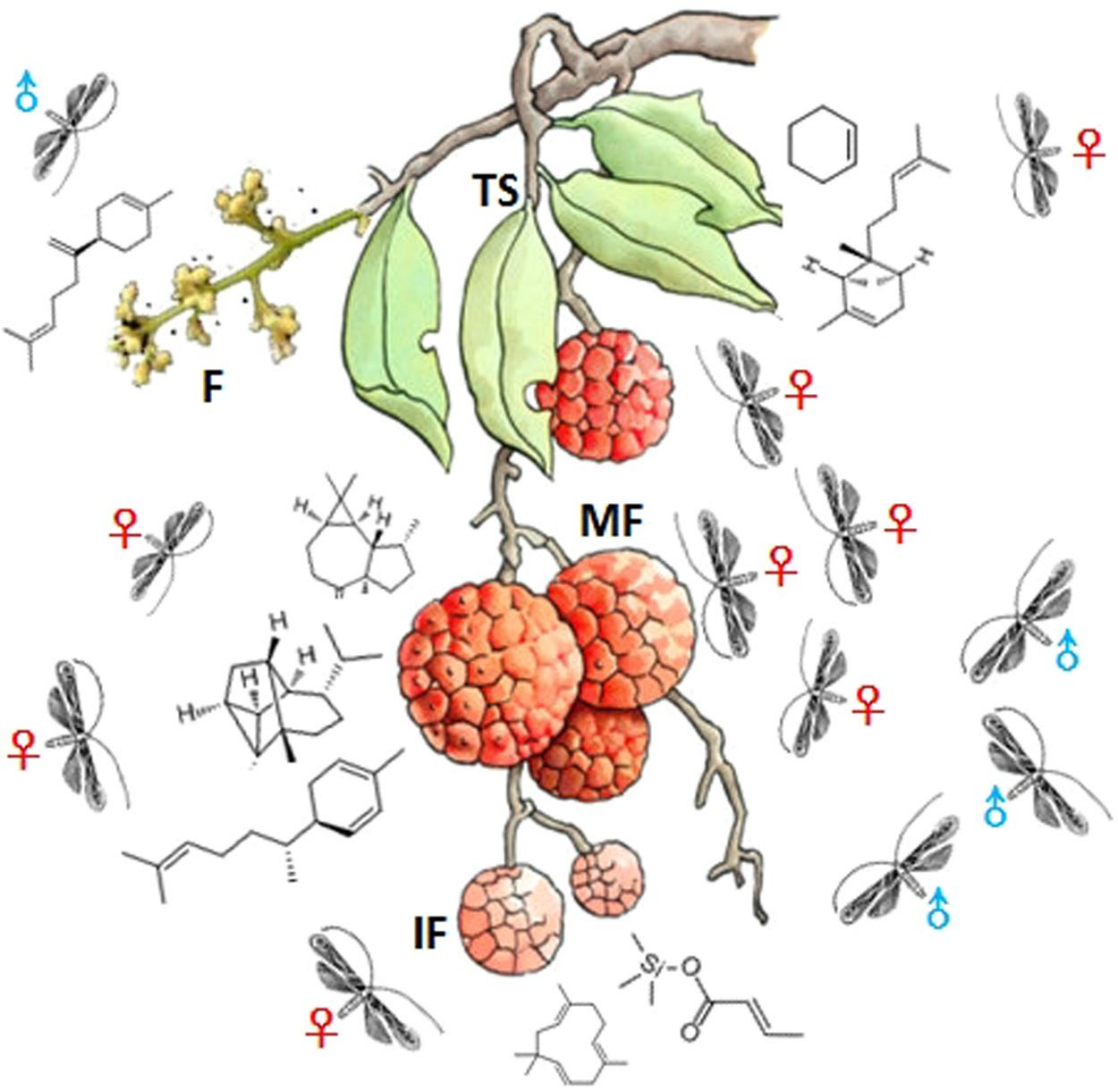
The host-specificity choices of *Conopomorpha sinensis*. F: flower; TS: tender shoots; IF: immature fruit; MF: mature fruit; chemical structures are different volatiles of litchi.

In conclusion, our studies indicate that *C. sinensis* has evolved physical and chemical mechanisms to locate a suitable host, thereby enabling it to survive and reproduce. Host plant volatiles have a significant effect on the oviposition preference of adult female *C. sinensis*, and the analysis and identification of these volatiles afford many valuable basal data and candidate volatiles, which are key factors in the choice of *C. sinensis* to oviposit on litchi (Fig. 5). To take advantage of the ability of host plant volatiles to influence directional selection in *C. sinensis*, it is necessary to determine the single and composite compounds of host volatiles that exert the greatest attractant activity to *C. sinensis*. Future work will require the implementation of electroantennogram (EAG) response measurements, wind tunnel tests, field tests, and molecular tools to tackle *C. sinensis* infestation effectively.

## Methods

### Insects and litchi fruits

*Conopomorpha sinensis* Bradley for olfactory experiments were collected from the litchi orchard, Institution of Fruit Tree Research, Guangdong Academy of Agricultural Sciences, Guangzhou, China. They were reared on litchi fruit in a climate room (26 ± 1 °C; 14 h Light:10 h Dark; 60**–**80% RH). Females and males were identified and separated according to their reproductive organs under a stereomicroscope.

Fresh fruits of the litchi cultivar Feizixiao were chosen as the basic material, since it is the cultivar most seriously affected by *C. sinensis*^15^. The litchi flower, tender shoot, fruit, and the fruit pericarp, pulp, seed were separated for experiments.

### *C. sinensis* attraction to the surfaces of host plants and non-host plants Attraction of *C. sinensis* to the surfaces of host plants

Ten mature litchi fruits were wrapped individually in either flat paper, convex paper, or concave paper; controls were not wrapped in paper. The fruit were then placed in the same insect rearing cage (45 cm × 45 cm × 50 cm). Twenty 2-day-old male and female adults of *C. sinensis* (♀:♂ = 1:1) were placed in one cage for the oviposition experiment with three replicates; every insect was used for once in the test. After 48 h, the egg numbers oviposited by females from each treatment was recorded.

### Attraction of *C. sinensis* to host plants and non-host plants

Ten each of the following were wrapped in convex paper: mature litchi fruits, fruits of a non-host plant (*Prunus salicina* Lindl, a kind of plum, the same size as litchi fruits), and non-host (red plastic balls of similar dimensions to litchi fruits). The wrapped fruits and plastic balls were placed in the same insect rearing cage (dimensions as above). The control consisted of the same number of mature litchi fruits, unwrapped. The oviposition test with three replicates were recorded as described above.

### Chemical choice behavior of *C. sinensis* to host plants Taxis reaction of *C. sinensis* to different tissues of litchi

The taxis reaction of *C. sinensis* to four different litchi tissues (flower, tender shoots, immature fruit, and mature fruit) was studied using the H-type olfactometer method of Kudon^25^ and Xian *et al*.^13^. The olfactometer is in the form of letter H and made of plexiglass. It had two cylindrical channels (Height: 30 cm, Diameter:10 cm) on each side, and the channels plus a cylindrical bioassay chamber (Length: 30 cm, Diameter: 3 cm) composed the olfactometer proper. Insects were placed in the central entry of the bioassay chamber and their activity to which side were recorded in the olfactometer. One side of the H-type olfactometer contained tender shoots, immature fruit, or mature fruit; the other side of the olfactometer contained nothing and was a blank control. Host selection behaviors were observed and recorded 1 hour after an insect was placed in the H-type olfactometer. Fifty male and female adult *C. sinensis* were tested in the experiment, which was repeated three times. Every insect was used for once in the test. The number of *C. sinensis* showing a taxis reaction to the different litchi tissues was recorded and the attraction rate was calculated.

### Oviposition preference of female *C. sinensis* on different tissues of litchi fruit

The oviposition preference of female *C. sinensis* was tested in an insect rearing cage (45 cm × 45 cm × 50 cm). Fifty 2-day-old male and female adult *C. sinensis* (♀:♂ = 1:1) were placed in the cage and fed with 5% honey, soaked into cotton. All tissues were wrapped in convex paper respectively. The cage was filled respectively with three litchi pericarps, pulps, seeds, and blank controls at random positions within the cage. Each experiment was performed in triplicate. The culture environment was at 26 ± 1 °C, 14 h:10 h (Light: Dark) photoperiod, and 60**–**80% RH. The number of ovipositing female *C. sinensis* was recorded after 48 h.

### Analysis of volatiles from different litchi tissues using HS-SPME-GC-MS Headspace solid-phase microextraction (HS-SPME)

Fresh litchi fruits were put into a 500 ml extraction bottle and sealed. The SPME conditions were further optimized by careful selection of the fibers, the extraction time, and the desorption time according to Bianchin *et al*.^47^. After 1 h microextraction, the extraction fiber was placed into the instrument for GC-MS analysis. The temperature for SPME fiber desorption was 200 °C with 3 min sample injection.

### GC-MS analysis

A gas chromatograph (GC-8000^Top^, Finnigan, USA) equipped with a gas chromatographic column (30 mm × 0.25 mm × 0.25 μm) was used during the study. The injector temperature was set at 240 °C and operated in split mode with a flow rate of 10 ml min^−1^. Ultrapure helium at 1 ml min^−1^ was used as the carrier gas. The oven temperature program was as follows: 40 °C (held for 4 min), 10 °C min^−1^ to 240 °C (held for 3 min). MS detection was performed under the following conditions: ionization source at 230 °C, and the ionization mode was electron impact with electron energy of 70 eV, 350 v. The mass spectrometer was operated in total scan mode over a m/z range of 35**–**350 amu. The analytes were univocally characterized on the basis of their retention times and their mass-to-charge ratios.

### Statistical analysis

The attraction rate formula of taxis reaction of *C. sinensis* to ifferent tissues of litchi = (number attracted by the treatment - number attracted by the control)/(n = 50) × 100%.

Statistical analysis was performed using SPSS Statistics 22 software (IBM Corp., Armonk, NY, USA). One-way ANOVA with a post hoc LSD test was used. Results were considered statistically significant when *p*-values were <0.05.

The chemical compounds of different litchi tissue volatiles were determined by analyzing and comparing the mass spectra using the U.S. National Institute of Standards and Technology (NIST) spectrum library, and the retention times from standard mass spectrometry.
